# Genome sequences of six clinical isolates of *Candida parapsilosis* exhibiting different degrees and temporal regulation of biofilm formation

**DOI:** 10.1128/mra.01300-24

**Published:** 2025-08-11

**Authors:** Sulman Shafeeq, Srisuda Pannanusorn, Jacques Dainat, Ali Dadvar, Christian Tellgren-Roth, Bengt Sennblad, Björn Nystedt, Ute Römling

**Affiliations:** 1 Department of Microbiology, Tumor and Cell Biology, Karolinska Institutet 27106, Stockholm, Sweden; 2 Department of Biotechnology, Faculty of Science and Technology, Thammasat University, Pathum Thani, Thailand; 3 Department of Medical Biochemistry Microbiology and Genomics, National Bioinformatics Infrastructure Sweden, Science for Life Laboratory, Uppsala University 8097, Uppsala, Sweden; 4 Science for Life Laboratory, Department of Immunology, Genetics and Pathology, Uppsala University 8097, Uppsala, Sweden; 5 Department of Cell and Molecular Biology, National Bioinformatics Infrastructure Sweden, Science for Life Laboratory, Uppsala University 8097, Uppsala, Sweden; University of California Riverside, Riverside, California, USA

**Keywords:** *Candida parapsilosis*, PacBio RSII system, biofilm formation, clinical isolates

## Abstract

*Candida parapsilosis* is a major pathogen causing central venous catheter–associated bloodstream infections with biofilm formation as virulence factor. We sequenced the genomes of six *C. parapsilosis* isolates from bloodstream infections displaying no, low, and high biofilm under conditions mimicking the clinical setting.

## ANNOUNCEMENT

Bloodstream infection caused by *Candida* species is a primary cause of morbidity and mortality in intensive care units worldwide (1–9). Thereby, the environmental species *C. parapsilosis* is the second/third most frequently isolated species (1, 10) causing superficial to invasive infections (1, 4). The ability to grow in parenteral nutrition with high glucose to form biofilms on catheters and high transmissibility contribute to the infection potential (11–13).

**Fig 1:**
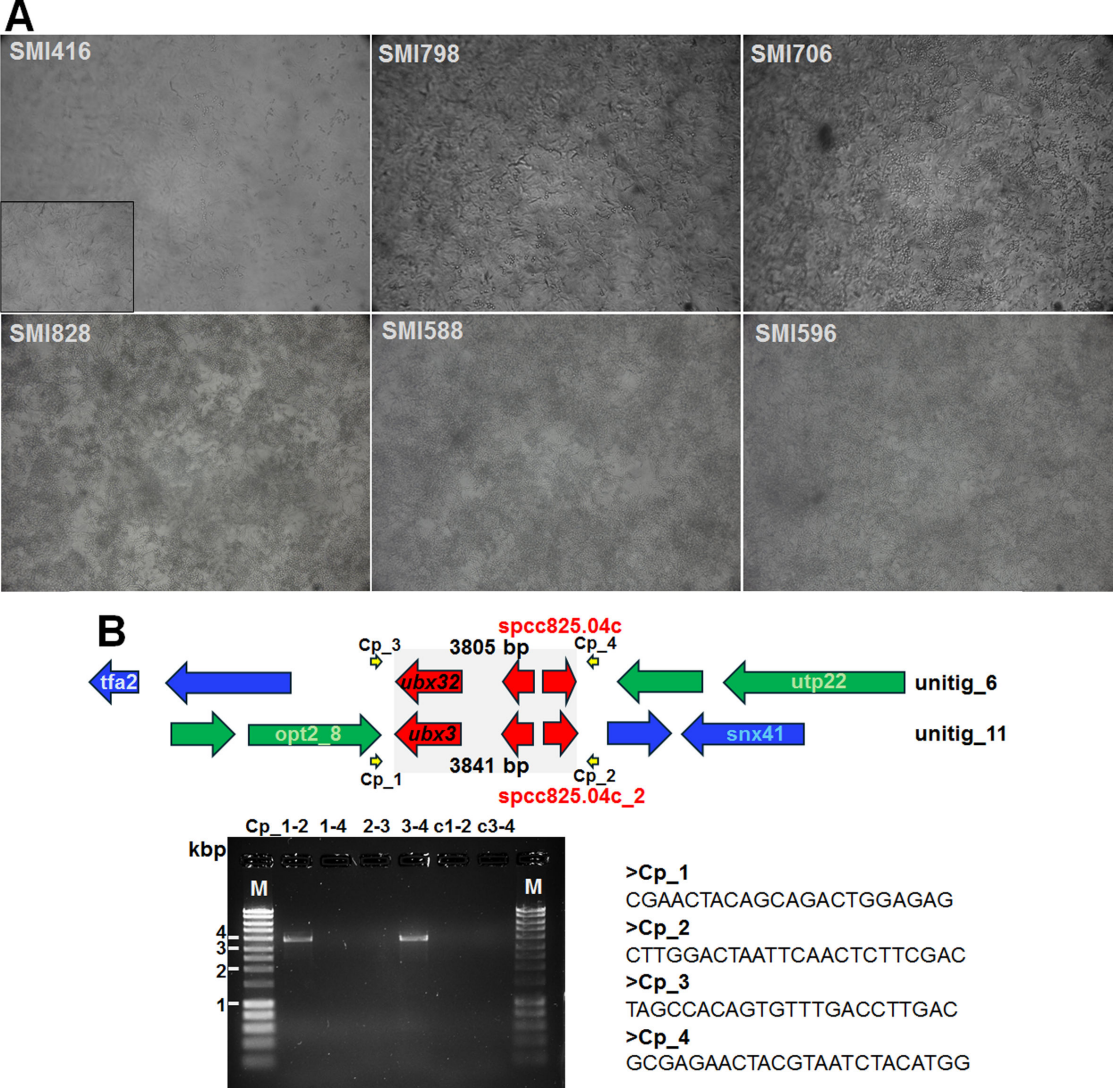
Biofilm formation of six *C. parapsilosis* isolates at 48 h on silicon pads developed in YNB medium with10% glucose mimicking parenteral solution as documented by light microscopy (**A**) with no, SMI416 (inset, silicon pad incubated with uninoculated medium), SMI798; low, SMI706; and high, SMI588, SMI596, SMI828, biofilm-forming ability (15); (**B**) translocation observed between *C. parapsilosis* SMI828 and CDC317 encompassing a 3,654 bp sequence duplication with 98.91% identity (gray box) in the two contigs in both strains. The translocation has been experimentally verified by PCR in SMI828 using the indicated primers located outside the repetitive sequences. Green and blue arrows indicate the location of genes on CDC317 contigs 005806 and 006372, respectively. Empy arrows/no text indicate gene of unknown function.

Here, we report whole-genome sequencing of six Swedish *C. parapsilosis* bloodstream isolates (14–16) with no (SMI 416, SMI 798), low (SMI 706 with high initial adherence), and high (SMI 588, SMI 596, SMI 828 [hospital outbreak strain (12)]) biofilm-forming ability (Fig. 1A).

Strains maintained at −80°C were plated on YPD (1% yeast extract, 2% peptone, and 2% glucose) agar plates and incubated at 37°C for 48 h. Subsequently, isolates were grown overnight in 50 mL YPD medium at 37°C with shaking, and genomic DNA was isolated using the Genomic-tip 500/G columns (QIAGEN) following the manufacturer’s instructions. RNA contamination and DNA integrity were investigated on a 0.5% agarose gel (1×TAE buffer), and quantity and purity were assessed by 260/280 and 260/230 ratios measured by NanoDrop 2000c (Thermo Scientific). Genomic DNA was sheared with Megaruptor 2, removing fragments below 3 kbp with SPRI beads. SMRTbell libraries prepared at the National Genomics Infrastructure (Science for Life Laboratory, Uppsala) according to the manufacturer’s instructions were sequenced in SMRT cells on the PacBio RS II system.

The quality of reads was evaluated with FastQC (17), InSeqt (http://grabherr.github.io/InSeqt/), and Kraken ((18), contamination). *De novo* genome assembly was performed by HGAP3 (Hierarchical Genome-Assembly Process)/HGAP4 (for SMI 706) algorithms from the PacBio SMRT tools (19). The assemblies were further evaluated using QUAST (20) to assess basic statistics and BlobTools (21) to again identify contaminations.

Annotation of each individual genome with three ab-initio tools used transcriptional data from RNA-sequencing experiments (differential gene expression in early versus late biofilms by the high spider-like biofilm-forming yeast *Candida parapsilosis* SMI828, by S.S. Manfred Grabherr, B.S., B.N., U.R., in preparation) and reviewed proteins from UniProt/Swiss-Prot (downloaded 2016-08 (22, 23)). Reads from RNA-sequencing were mapped using *tophat2* (v.2.0.9) (24) and assembled with the *stringtie* package (v.1.2.2) (25). The three ab initio tools, GeneMark-ET v.4.3 (26), Augustus (integrates transcriptional and protein data [Augustus (27)]), and SNAP (pure ab initio (28)) were run within the *Maker* v.3.0 package (29) to combine annotations for a comprehensive gene build. Translated coding sequences were searched with BlastP against the UniProt/Swiss-Prot reference data set (22, 23) and run against InterProScan v.5.7-48 (30) to retrieve InterPro, PFAM, Gene Ontology (GO), MetaCyc, UniPathway, KEGG, and Reactome assignments. The data output was parsed using the AnnIE annotation tool (31). Mitochondrial genomes were also annotated using the MFannot server (32).

The six *de novo* assembled and annotated CTG clade *C. parapsilosis* genomes are larger than the *C. parapsilosis* CDC317 reference genome (eight chromosomes of 12,998,393 bp in total and a mitochondrial genome of 31,781 bp) (Table 1). Comparative analysis indicated the presence of a large chromosomal translocation in SMI 828 (and all other sequenced genomes) compared to CDC317 (Fig. 1B).

**TABLE 1:** Basic characteristics of *de novo* assembled and annotated genomes of the six Swedish *C. parapsilosis* isolates

Strains	GC-content %	Length [bp]	Coverage	#contigs	Largest contig [bp]	N50 [bp]	Number of raw reads	L50	Predicted genes
	High biofilm-forming strains								
SMI828	38.7	13,575,533	206.70	38	1,981,830	1,105,301	229,353	5	6,128
SMI588	38.7	13,478,561	144.05	30	2,092,877	1,400,889	180,203	4	6,160
SMI596	38.7	13,244,759	131.82	21	3,027,088	2,087,741	174,490	3	6,052
	No biofilm-forming strains								
SMI798	38.7	13,395,816	131.28	28	3,025,112	2,006,230	169,821	3	6,070
SMI416	38.7	13,414,442	133.33	33	3,031,530	1,402,579	168,108	4	6,137
	Low biofilm-forming strains								
SMI706	38.5	13,322,571	327	29	1,990,489	1,465,361	203,964	4	6,038
	Reference strain								
CDC317	38.5	13,030,174	9.2	8	n.r.^*a*^	2,100,000	3,023,470	3	5,827

^*a*^ n.r., not relevant.

## Data Availability

This Whole Genome Shotgun project has been deposited in DDBJ/ENA/GenBank under the project ID PRJEB73879/ERP158620 (https://www.ebi.ac.uk/ena/browser/view/PRJEB73879). The version described in this paper is the first version of CAXIDQ010000000, CAXIDR010000000, CAXIDS010000000, CAXIDT010000000, CAXIDU010000000 and CAXIDV010000000. RNA sequencing data have been deposited in DDBJ/ENA/GenBank under the project ID ERP173917.
